# Influenza M2 virus-like particle vaccination enhances protection in combination with avian influenza HA VLPs

**DOI:** 10.1371/journal.pone.0216871

**Published:** 2019-06-27

**Authors:** Hae-Ji Kang, Ki-Back Chu, Dong-Hun Lee, Su-Hwa Lee, Bo Ryoung Park, Min-Chul Kim, Sang-Moo Kang, Fu-Shi Quan

**Affiliations:** 1 Department of Biomedical Science, Graduate School, Kyung Hee University, Seoul, Republic of Korea; 2 Institute for Biomedical Sciences, Georgia State University, Atlanta, GA, United States of America; 3 Department of Medical Zoology, Kyung Hee University School of Medicine, Seoul, Republic of Korea; 4 Medical Research Center for Bioreaction to Reactive Oxygen Species and Biomedical Science Institute, School of Medicine, Graduate school, Kyung Hee University, Seoul, Republic of Korea; University of Iowa, UNITED STATES

## Abstract

Despite the ability to induce a broad range of cross protection, M2e5x virus-like particles (VLPs) alone provide limited vaccine efficacy and confer low efficacy of protection against highly pathogenic avian influenza virus (HPAIV) infection in chickens. Avian influenza hemagglutinin (HA) has been a major antigenic target that enhances humoral immunity, but the efficacy of avian HA-based vaccines against HPAIV needs to be further improved. In this study, we evaluated the vaccine efficacy induced by combination of conserved tandem repeat M2e5x VLPs and HA VLPs against an avian influenza virus. We found that combinatorial vaccine elicited higher levels of reassortant H5N1 (rgH5N1) virus-specific IgG, IgG1, IgG2a and IgA antibody responses compared to M2e5x VLPs in sera and lungs of mice. Combinatorial VLPs vaccination induced higher levels of CD8^+^ T cell and germinal center B cell responses in lung and spleen compared to M2e5x VLPs. Combinatorial VLPs vaccination showed significantly reduced inflammatory responses and lung viral loads upon rgH5N1 virus challenge infection, resulting in less body weight loss compared to M2e5x VLPs alone. These results indicate that immune responses to both M2e and HA might provide a strategy of vaccination inducing enhanced protection against avian influenza virus.

## Introduction

Every year, highly pathogenic and contagious avian influenza causes thousands of poultry deaths, resulting in severe economic losses to the poultry industry [1]. Poultry vaccination regimen for regulating highly pathogenic avian influenza virus (HPAIV) H5N1 has been implemented in several countries throughout the globe, but field trial results assessing its effectiveness remains unreported [2]. Avian influenza virus vaccination failures and the absence of effective protective immunity were also reported [2]. As such, there is a critical need to develop a more effective vaccine that prevents poultry from HPAI infection.

The extracellular domain of ion channel M2 (M2e) is highly conserved among influenza A viruses and has been reported to be a target for cross protection. M2e5x virus-like particles (VLPs) have been generated by genetically engineering a tandem repeat comprising highly conserved M2e epitope sequences (M2e5x) from multiple host-origin influenza viruses with influenza matrix protein 1. M2e5x VLPs vaccine showed significant improvement in cross-protection in mouse models [3–5]. M2e5x VLP vaccine containing a tandem repeat of M2e sequences (M2e5x) derived from human, swine, and avian origin influenza A virus provided cross protection against H1, H3, and H5 subtype influenza viruses in a mouse model [6]. However, vaccination with M2e5x VLPs alone was unable to protect chickens from HPAI infection, resulting in no protection [1]. There is an urgent need for developing highly immunogenic avian influenza vaccine.

Most conventional avian influenza vaccines are based on the immunity to hemagglutinin (HA) protein. The hemagglutinin protein is a major target of protective antibody response induced by vaccination. HA-based vaccines inducing strain-specific immunity could be effective in the poultry industry. However, several factors limit vaccine efficacy, including variability and fast mutation rates of HA antigens of the virus. Thus, one of the major challenges in the poultry industry is in the development of broad-spectrum avian influenza vaccine, a similar problem against human influenza. Adenovirus-based influenza A virus vaccine containing hemagglutinin (HA) protein of the A/Vietnam/1203/2004 (H5N1) protected domestic chickens from an intranasal challenge with VN/1203/04 [7]. Vaccination with virus-like particles containing HA proteins derived from three distinct clades of H5N1 viruses protects chickens from H5N1 and H5N8 influenza viruses [8]. Previous studies also reported enhanced cross protection by combination of M2e5x VLP and inactivated virus HA-based vaccination in mice or chickens [1,4]. Influenza virus HA or neuraminidase (NA) surface glycoproteins might have undergone point mutations (antigenic drifts) and genetic reassortments (genetic shifts) by mixing genomic segments from different viruses that results in a novel virus [9]. Thus, evaluating vaccine efficacy induced by combinatorial VLPs using M2e5x, as a potential candidate for a universal influenza vaccine and HA VLPs as a major target of protective antibody responses, would have significant impact.

In this study, we evaluated vaccine efficacy induced by combinatorial VLPs, which is a combination of two separate VLPs (one HA and another M2e5x), aiming to develop highly immunogenic vaccine. We found that combinatorial VLPs vaccination significantly reduced lung virus loads and inflammatory responses, providing better protection compared to M2e5x VLPs.

## Materials and methods

### Ethics statement

Animal experiment in this study was carried out under the guidelines set out by Kyung Hee University IACUC. The protocol was approved by the Committee on the Ethics of Animal Experiments of the Kyung Hee University (permit number: KHUASP(SE)-18-024). Immunization and bleeding were performed under mild anesthesia, which were induced and maintained with ketamine hydrochloride and xylazine. All efforts were made to minimize the number of animals used in the experiment as well as their suffering.

### Cells, viruses, animals, and antibodies

*Spodoptera frugiperda* Sf9 insect cells were used for production of recombinant baculovirus (rBV) and VLPs in serum-free SF900 II medium (Invitrogen, Carlsbad, CA, USA). VLPs were generated by infecting Sf9 cells with recombinant baculovirus (rBV) at multiplicity of infection (MOI) of 0.05. [10,11]. Madin-Darby canine kidney (MDCK) cells were grown and maintained in Dulbecco’s modified Eagle’s medium (DMEM) (Welgene, Daegu,Korea) supplemented with 10% fetal bovine serum, 1% penicillin and streptomycin. Avian influenza viruses A/Viet Nam/1203/2004 (rgH5N1) were propagated as previously described [12]. Inactivation of the purified virus was performed by mixing the virus with formalin at a final concentration of 1:4000 (v/v) as described previously [13]. Seven-weeks-old female BALB/c mice were purchased from KOATECH (Pyeongtaek, Korea). Horseradish peroxidase (HRP)-conjugated goat anti-mouse IgA, IgG, IgG1 and IgG2a antibodies were purchased from Southern Biotech (Birmingham, AL, USA).

### Generation of virus-like particles (VLPs)

A/Viet Nam/1203/2004 (rgH5N1) virus was inoculated into MDCK cells and then total viral RNA was extracted using RNeasy Mini kit (Qiagen). Total RNA was reverse-transcribed to cDNA using Prime Script 1st strand cDNA synthesis kit (Takara, Otsu, Japan) according to the manufacturer’s instructions. Avian influenza A/Viet Nam/1203/2004 (rgH5N1) HA gene was PCR-amplified with cDNA and primers containing restriction enzyme sites for cloning into the pFastBac plasmid expression vector (forward primer, 5- ATAT GGATCC ATGGA GAAAA TAGTG 3-; reverse primer, 5- ATAT AAGCTT TTAAA TGCAA ATTCT 3-; BamHI and HindⅢ sites are underlined). M1 gene from A/PR/8/34 was cloned as described previously[11]. HA and M1 gene nucleotide sequences were identical to the previously published sequences (accession no. KM186135.1, EF467824). Recombinant baculoviruses (rBVs) expressing HA or influenza M1 were generated as described [14]. Transfection of Sf9 cells with bacmid DNA using cellfectin II was performed followed by transformation of pFastBac containing HA or M1 with white/blue screening as described [14]. Afterwards, HA VLPs containing HA and M1 were produced in insect cells by co-infection with HA and M1 expressing rBVs as described [10,11]. VLPs were purified through a 20–30–60% discontinuous sucrose gradient at 30,000rpm for 1 h at 4°C as described [11]. M2e5x construct comprising heterologous tandem repeat of M2e peptides derived from human, swine, and avian origin influenza A viruses was previously described [3]. M2e5x VLPs were produced by co-infecting insect cells with rBVs expressing influenza M1 matrix protein and M2e5x as described previously [3]. Protein concentrations of the VLPs were measured using BCA Assay Kit (Thermo, USA). Two separate vaccines of M2e5x VLPs and HA VLPs were prepared independently and combined when immunizing mice.

### Characterization of HA VLPs and M2e5x VLPs

VLPs were characterized using western blot and ELISA. VLPs were separated on a polyacrylamide gel, transferred to PVDF membrane, then blocked with 5% skim milk. Membranes were probed with mouse serum collected at week 4 from BALB/c mice infected with avian influenza A/Viet Nam/1203/2004 (rgH5N1), anti-M1 monoclonal antibody (GA2B) (Abcam, Cambridge, MA) and anti-M2e monoclonal antibody (14C2) (Abcam, Cambridge, MA) overnight at 4°C. HRP-conjugated goat anti-mouse IgG was used as secondary antibody. Additionally, M2e5x VLPs were also confirmed by enzyme-linked immunosorbent assay (ELISA). M2e5x VLPs were coated using various concentrations and confirmed by sequentially incubating with anti-M2 monoclonal antibody and HRP-conjugated goat anti-mouse IgG.

### Immunization and challenge

Female, seven weeks old BALB/c mice were used. Groups of mice (n = 8) were intramuscularly (IM) vaccinated with HA VLPs (5ug) alone, combination VLPs (M2e5x VLPs 10μg + HA VLPs 5μg) or M2e5x VLPs (10μg) alone in 100μL at weeks 0 and 4. VLPs for immunization are suspended in PBS and naïve mice were given PBS only. The ratio of M2e5x with HA VLPs in combinatorial VLPs is 2:1, similar to previous studies [1,3,15,16]. At four weeks after boost immunization, mice were challenged with 7.5x10^2^ pfu (50 μL PBS) of mouse lethal dose (5xLD_50_) of reassortant rgH5N1 virus containing HA and NA derived from avian influenza A/VietNam/1203/2004 (H5N1) and the remaining 6 internal genes from A/PR8 H1N1 virus. After monitoring body weight and survival, half of the mice were sedated and sacrificed 4 days post-infection (p.i.) for lung and spleen collection. Mice that displayed greater than 25% loss in body weight were considered to reach the endpoint of death and euthanized.

### Influenza virus specific antibody responses and hemagglutinin inhibition (HAI) titers

The retro-orbital plexus puncture method was used to collect blood samples from mice at weeks 1 and 4 after prime and boost. To measure influenza-specific antibodies, flat bottom 96-well immunoplates (SPL Life Sciences, Korea) were coated overnight at 4°C with 100 μL of avian influenza A/Viet Nam/1203/2004 (rgH5N1) inactivated virus antigen at a final concentration of 4 μg/mL in 0.05M, pH 9.6 carbonate bicarbonate buffer per well. M2e-specific IgG antibody responses were also determined by using human influenza virus M2e peptide antigens as previously described [17]. 100 μL of serum samples (diluted 1:50, 1:150, 1:450 in PBST) were added to respective wells and plates were incubated for 1 h at 37°C. HRP-conjugated goat anti-mouse IgA, IgG, IgG1 and IgG2a antibodies diluted in PBS (100μL/well) were added for secondary antibody at 37°C for 1hr. HAI titers were determined using 0.5% chicken red blood cells and 4 HA units per well of rH5N2 virus as described previously[18].

### Lung viral titers, cytokine, and antibody secreting cells (ASC)

The viral titers in lung extracts were measured using MDCK cells as previously described[10]. Cytokines interferon-gamma (INF-γ) and interleukin-6 (IL-6) ELISA were performed as described previously [11]. BD OptEIA Set (BD Biosciences) were used for detecting cytokine levels in lung extracts. For ASC assays, 96-well culture plates were coated with avian influenza A/Viet Nam/1203/2004(rgH5N1) inactivated virus (4ug/ml) overnight as previously described [18–20]. Spleen and lung cells were added to coated plates after blocking and incubated for 2 h at 37°C with 5% CO2 as described previously[11]. Cells from plates were washed out and incubated with horseradish peroxide (HRP)-conjugated goat-anti mouse antibodies (IgG) for 1 h at 37°C. Secreted antibody levels from spleen and lung cells were determined after 5 days *in vitro* culture.

### Cell preparation in lung

Cells were prepared as indicated [6]. Cell suspensions were filtered through a cell strainer and the resulting filtrate was centrifuged at 4°C, 6000RPM for 10min. Supernatants were stored at -80°C until use. Cell pellets were resuspended in 44% Percoll solution and over layered on top of 67% Percoll prior to centrifugation at 3000rpm, 4°C for 20min. Cell layer near the interface between the two density gradients were collected and centrifuged for 10min at 4°C, 6000 rpm.

### Immune cell responses by flow cytometry

The activity of immune cells in the spleen and lung was analyzed by flow cytometry as described previously [21]. Percentages of T cells (CD4^+^, CD8^+^) and B cell (germinal center) from splenocytes and lung cells of mice were analyzed by flow cytometry 4 days post-challenge as described previously [6,22]. Splenocytes and lung cells (1×10^6^ cell/mL) in staining buffer (2% bovine serum albumin and 0.1% sodium azide in 0.1 M PBS) were incubated at 4°C for 15 min with Fc Block (BD Biosciences, CA, USA). For surface antigen staining, cells were incubated with fluorophore-conjugated antibodies (CD3e, CD4, CD8, B220, GL7; BD Biosciences) at 4°C for 30 min. Splenocytes and lung cells were washed with staining buffer and fixed with 4% paraformaldehyde for 30 min at 4°C before acquisition using a BD Accuri C6 Flow Cytometer (BD Biosciences). Data were analyzed using C6 Analysis software (BD Biosciences).

### Statistical analysis

All parameters were recorded for individuals within all groups. Data sets were compared using non-parametric one-way analysis of variance (ANOVA) Kruskal–Wallis test and the ANOVA with multiple comparison test of PC-SAS 9.3 (SAS Institute; Cary, NC, USA). A p-value < 0.05 was considered statistically significant.

## Results

### Characterization of HA VLPs and M2e5x VLPs

**Fig 1:**
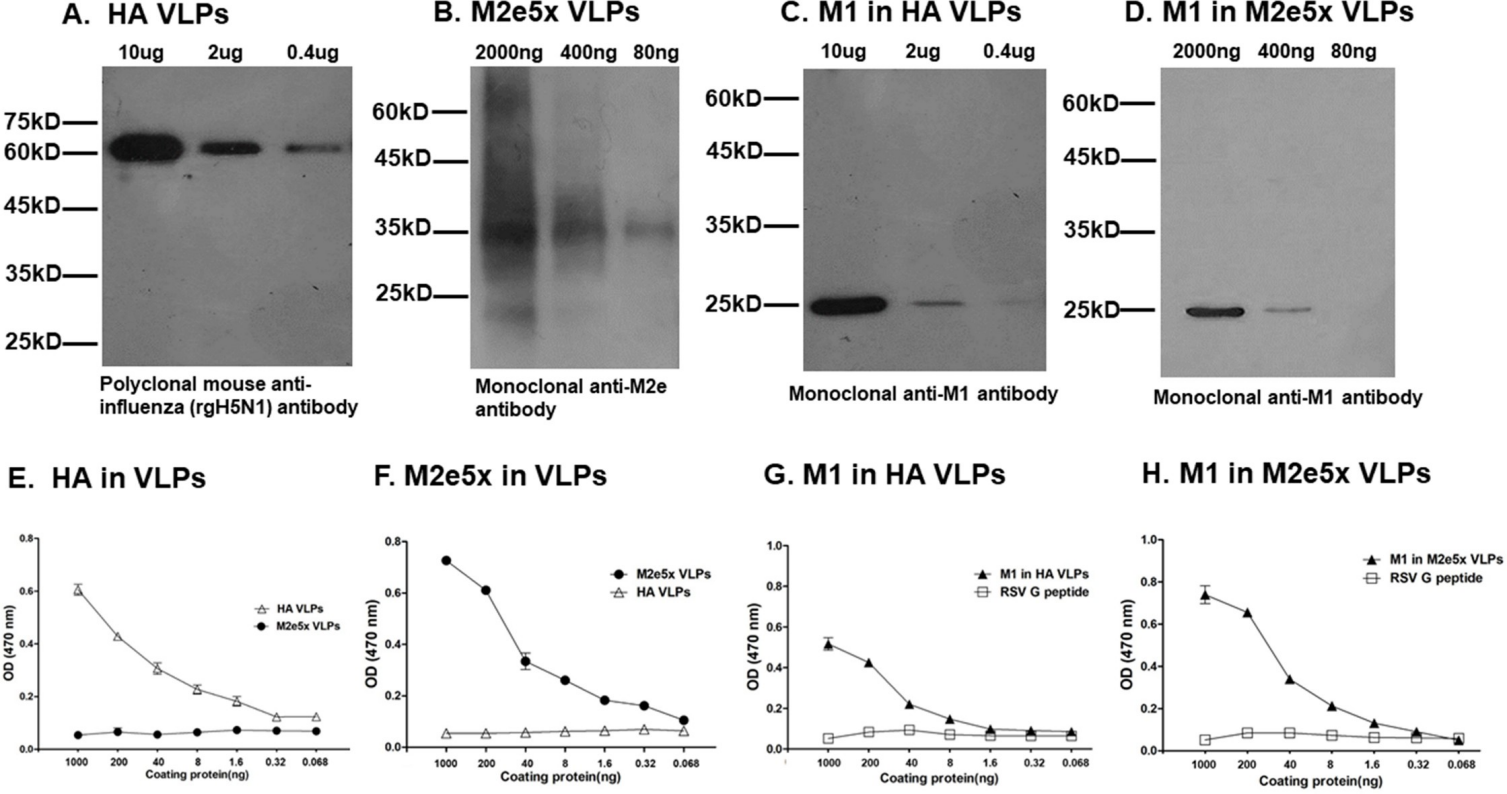
Identification of characterization of HA VLPs and M2e5x VLPs. Influenza HA VLPs, M1 VLPs (10ug, 2 ug, 0.4 ug) and M2e5x VLPs (2000ng, 400ng, 80ng) were loaded per lane and confirmed using western blot. Polyclonal mouse anti-influenza (rgH5N1) antibody was used to detect HA VLPs (62kDa, A) and M2e5x VLPs (37kDa, B) and M1 VLPs (28kDa, C, D) were probed with monoclonal anti-M2e antibody and anti-M1 monoclonal antibody, respectively. HA proteins in VLPs (Fig 1E), M2e5x proteins in M2e5X VLPs (F) and M1 proteins in VLPs (G, H) in HA or M2e5x VLPs were determined by ELISA.

VLPs expressing HA or M2e VLPs with influenza M1 were confirmed by western blots and ELISA using HA, M1, and M2e specific antibodies. HA (62kDa) (Fig 1A and 1E), M2e5x VLPs (37kDa) (Fig 1B and 1F), influenza M1 (28kDa) in HA VLPs (Fig 1C and 1G) and influenza M1 in M2e5x VLPs (Fig 1D and 1H) were observed at high levels. All of the aforementioned VLPs were detected using anti-influenza polyclonal antibody, anti-M1 monoclonal antibody and anti-M2 monoclonal antibody, each respectively.

### VLPs combination induced higher levels of avian influenza virus-specific antibody responses compared to M2e5x VLPs

**Fig 2:**
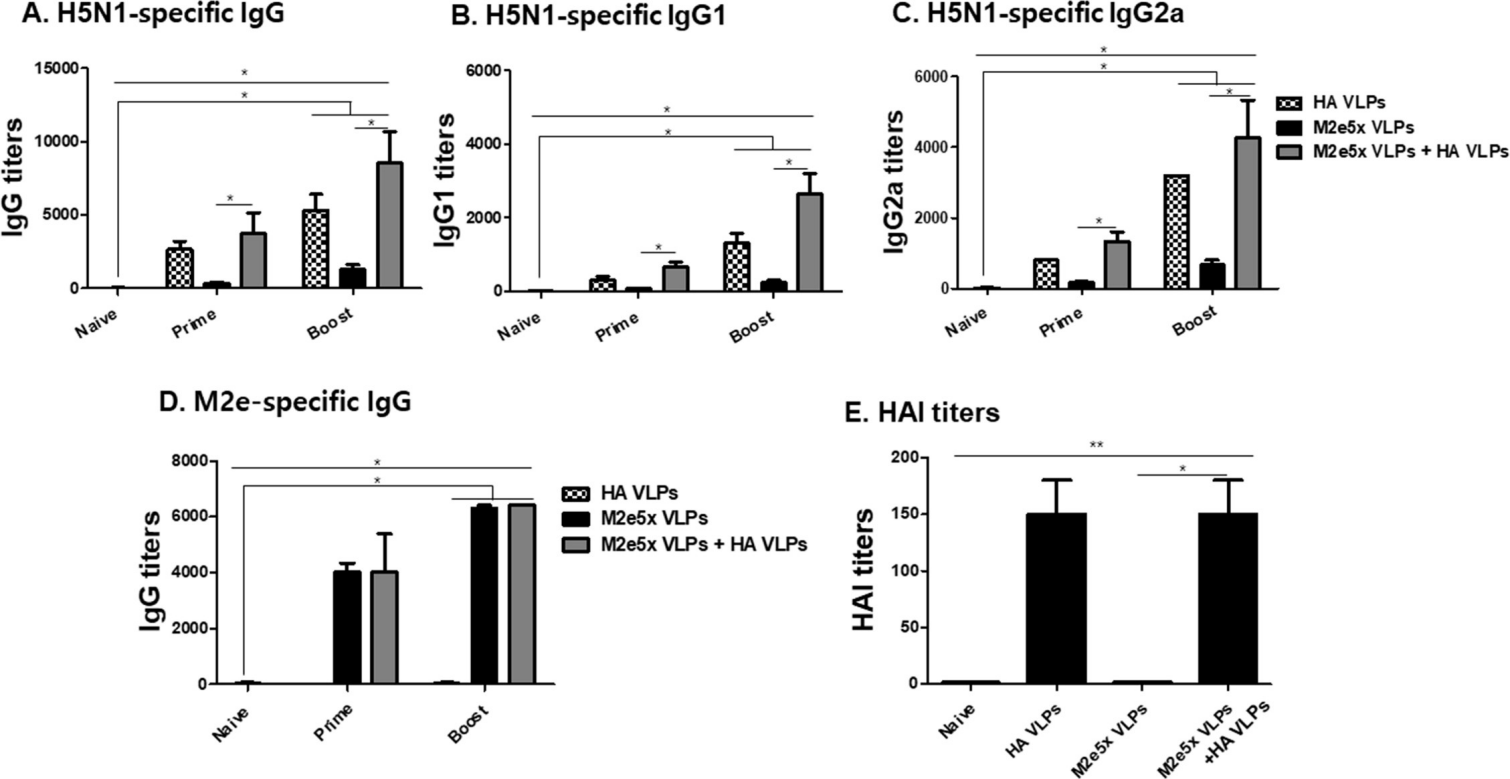
Antibody response in sera. Mouse sera collected from immunized mice were used to determined antibody responses. H5N1—specific IgG, IgG1 and IgG2a antibody responses and M2e - specific IgG antibody responses were determined. Significantly higher levels of influenza virus—specific IgG (A, * P < 0.05), IgG1 (B, * P < 0.05) and IgG2a (C, *P < 0.05) antibody responses after boost were found compared to prime and naïve. Higher levels of IgG and IgG2a antibody responses were found compared to IgG1 in combinatorial VLPs group compared to M2e5x VLPs. Similar levels of M2e - specific IgG antibody responses (D, *P < 0.05) were found in M2e5x VLPs and combinatorial groups. Higher levels of HAI titers against rH5N1 virus in sera were found in combinatorial and HA VLPs groups (**P < 0.05). Data are expressed as mean ± SD.

The levels of influenza-specific IgG, IgG1, and IgG2a antibody responses and M2e-specific IgG antibody responses in sera after prime and boost were measured. As shown in Fig 2, the combinatorial VLPs (M2e5x VLPs + HA VLPs) immunized group showed higher levels of influenza-specific IgG (Fig 2A), IgG1 (Fig 2B) and IgG2a (Fig 2C) antibody responses compared to M2e5x VLPs and HA VLPs (*P < 0.05). The M2e5x VLP and combination VLP (M2e5x VLPs + HA VLPs) groups induced similarly high levels of M2e specific IgG antibody responses (Fig 2D). Significantly higher levels of IgG2a antibody responses were found compared to IgG1 antibody responses in combinatorial VLPs group (*P < 0.05). To investigate immune correlates for predicting protection, HAI titers in immune sera were determined (Fig 2E). As expected, combinatorial VLPs vaccination showed similar level of HAI titers to HA VLPs whereas no HAI titers were found in M2e5x VLPs vaccination (Fig 2E).

### Combinatorial VLPs induced higher level of IgG antibody secreting cell (ASC) responses in spleen and lung, and IgG, IgG1, IgG2a and IgA antibody responses in lungs

**Fig 3:**
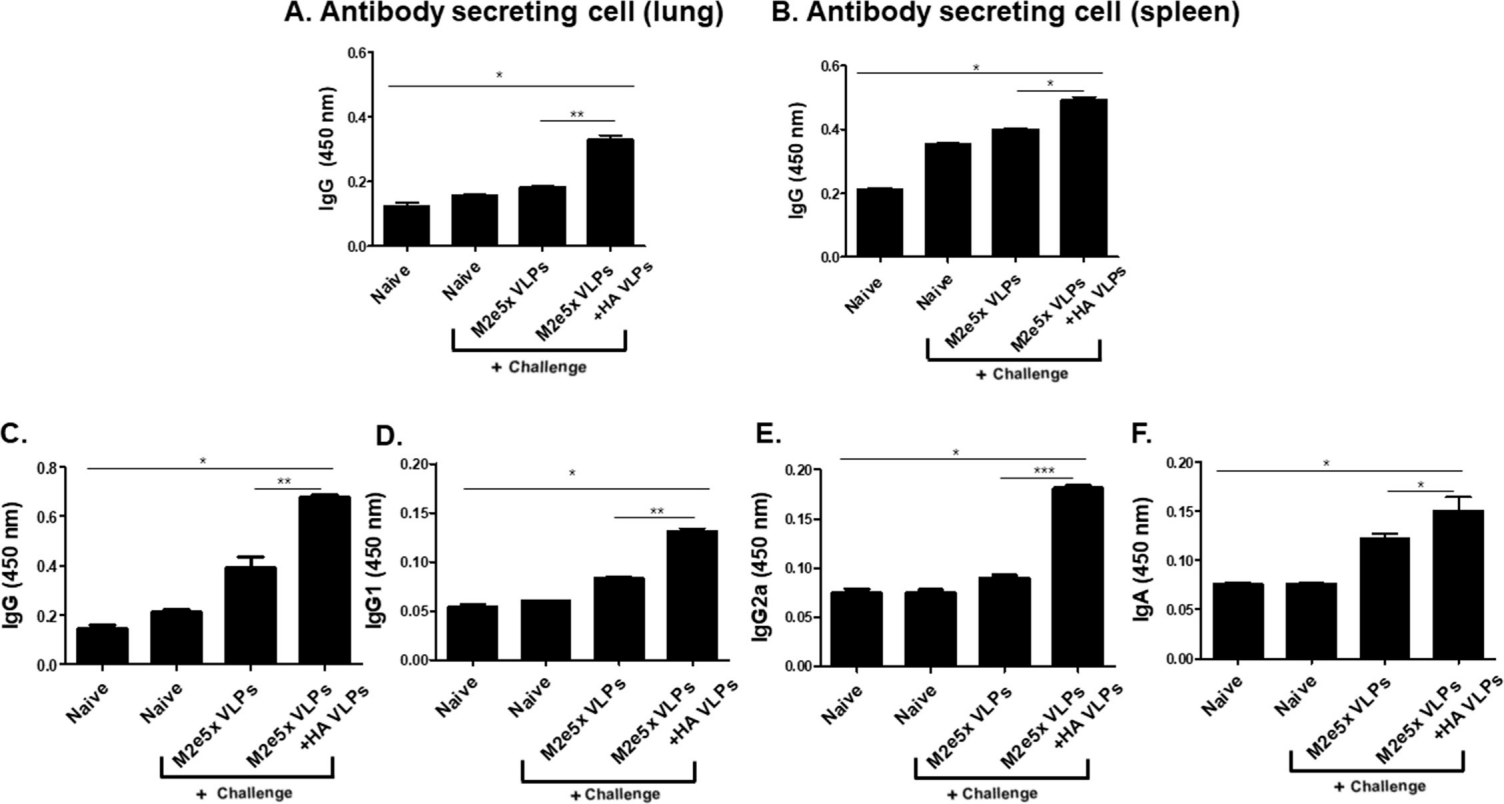
Antibody secreting cell responses (ASCs) from lung and spleen and antibody responses in lungs. Immunized mice were challenge infected with lethal dose of A/Viet Nam/1203/2004 (rgH5N1) virus. At day 4 post-challenge infection, ASCs responses were measured from splenocytes (A) and lung cells (B). Antibody responses from lung extracts were also measured IgG (C), IgG1 (D), IgA (E) and IgG2a (F). Data are expressed as mean ± SD (*P<0.05).

A goal of vaccination is to generate long-lived memory B cells that can rapidly differentiate into ASCs upon re-exposure to antigens [18,19,23]. To determine influenza virus-specific antibody secreting cell responses, cells from the spleens and lungs were collected and incubated 5 days in 96 well plate coated with avian influenza A/Viet Nam/1203/2004(rgH5N1) inactivated virus. As seen in Fig 3A and 3B, significantly higher levels of avian influenza-specific antibodies were secreted from the splenocytes (Fig 3A) and lung cells (Fig 3B) in combinatorial VLPs group compared to those in naive mice and M2e VLPs group. ASCs determination from lung cells has never been reported. Interestingly, higher levels of IgG, IgG1, IgG2a and IgA antibody responses were found from lung extract in combinatorial VLPs group compared to those in M2e5x VLPs (Fig 3C–3F). Compared to ASCs produced in spleens, ASCs in lungs showed higher levels of IgG secreting antibody responses. These results indicate that antibody secreting cell responses from lung cells could be a way to determine antibody response induced by vaccination.

### Combinatorial VLPs induced higher level of cellular immune cell response

**Fig 4:**
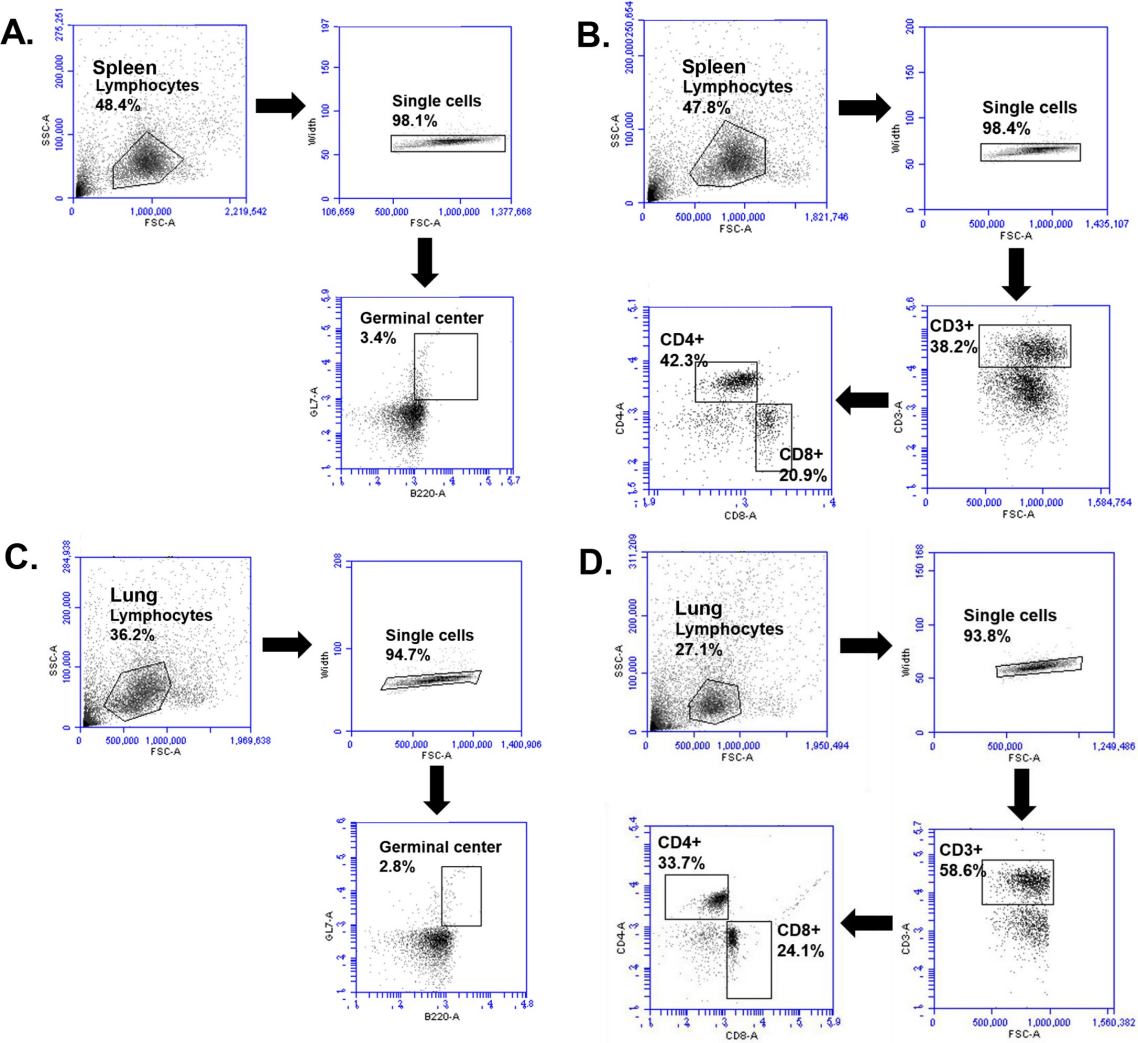
Gating strategy panels. (A) Flow cytometry plots showing the gating strategy to identify germinal center cells in spleens. (B) Flow cytometry plots showing the gating strategy to identify CD4^+^ and CD8^+^ T cell in spleens. (C) Flow cytometry plots showing the gating strategy to identify germinal center cells in lung cells. (D) Flow cytometry plots showing the gating strategy to identify CD4^+^ and CD8^+^ T cell in lung cells.

**Fig 5:**
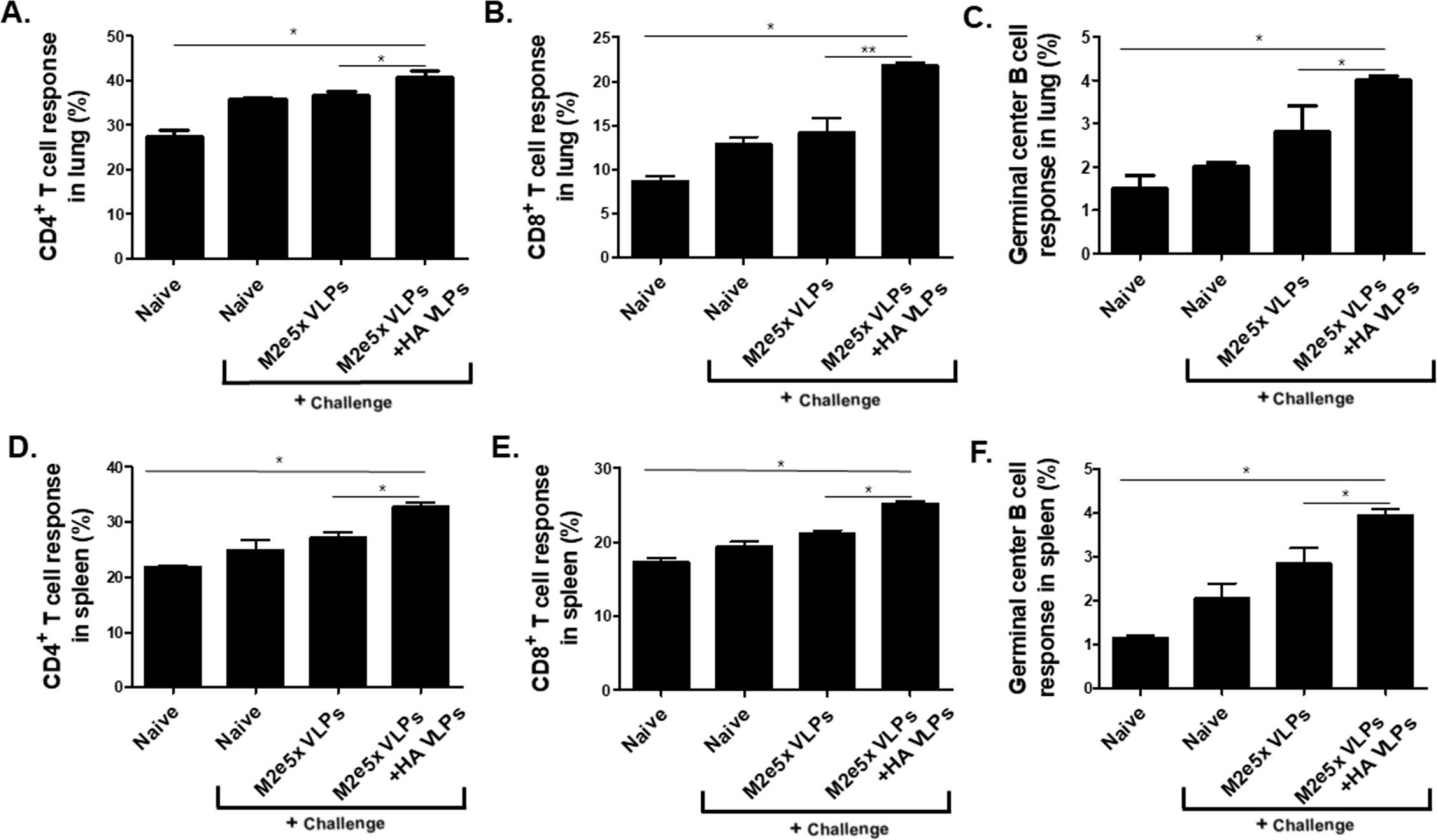
Combinatorial VLPs vaccine induces high levels of CD4^+^, CD8^+^ T cell and germinal center B cell responses after challenge infection. CD4^+^ T cells (A and D), CD8^+^ T cells (B and E) and germinal center (GC) B cell (C and F) in lung and spleen were analyzed by flow cytometry at 4 days after challenge with A/Viet Nam/1203/2004 (rgH5N1) virus. Percentages of cell populations in each. Higher populations of CD4^+^, CD8^+^, and GC B cell were detected in combinatorial VLPs group compared to M2e5x VLPs or Naïve + Challenge group (*P < 0.05). Data are expressed as mean ± SD.

To determine cellular immune cell response, gating strategies for CD4^+^ and CD8^+^ T and germinal center B cell populations were determined (Fig 4). Overall, as shown in Fig 5, combinatorial VLPs showed higher levels of immune cells responses, with particularly high levels for germinal center B cell and CD8^+^ T cell in the lung (Fig 5B and 5C) and spleen (Fig 5E and 5F). These results indicate that highly active immune cells confer increased protection required for virus inhibition.

### Combinatorial VLPs significantly reduced lung inflammatory responses

**Fig 6:**
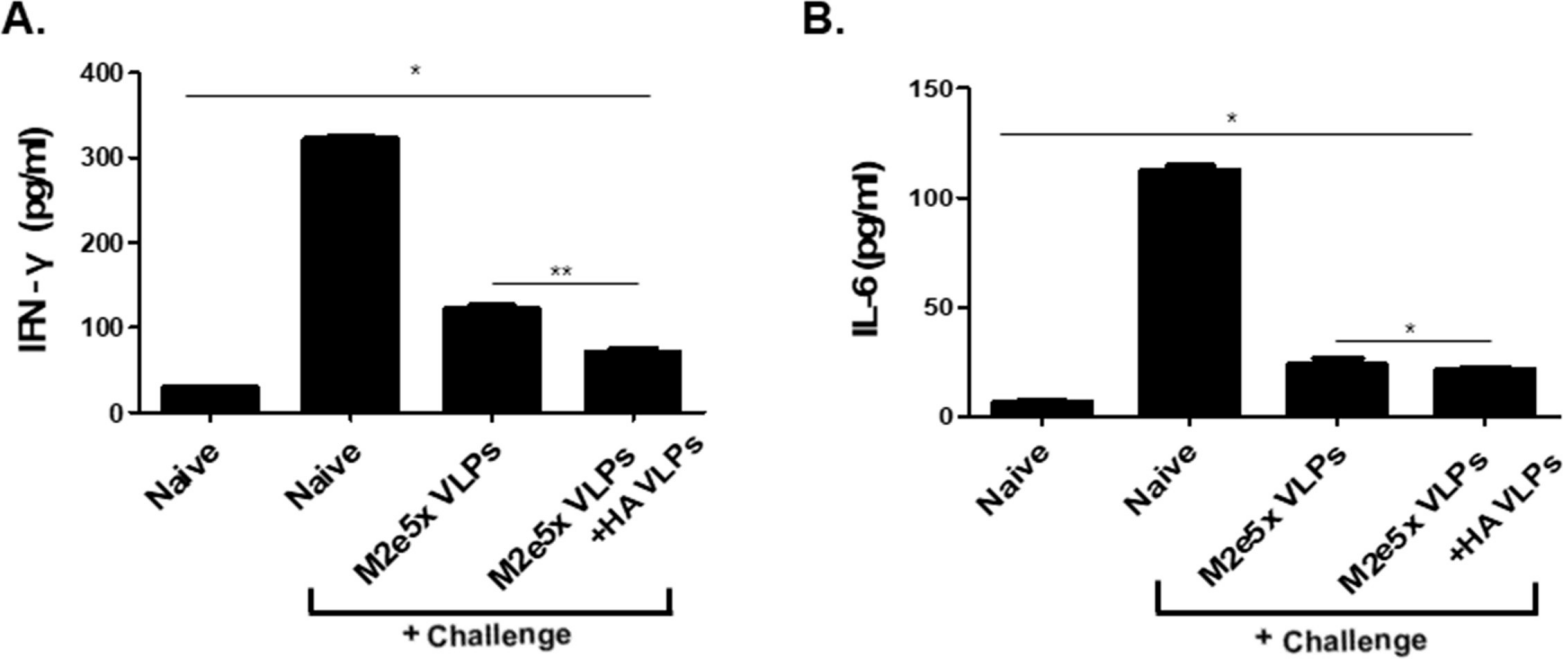
Inflammatory responses. Levels of inflammatory cytokines IFN-γ (A) and IL-6 (B) were also determined in the lung extracts at day 4 after challenge with A/Viet Nam/1203/2004 (rgH5N1) virus. Combinatorial group showed significantly lower levels of IFN-γ compared to M2e5x VLPs alone group and Naïve + Challenge group (*P < 0.05). Data are expressed as mean ± SD.

Influenza virus infection can induce high levels of proinflammatory cytokines in lungs, causing tissue damage which may lead to death[10]. High levels of inflammatory cytokines including IL-6 and IFN-γ gamma were shown to be induced by infection with highly pathogenic influenza viruses and high lung viral titers, causing severe inflammatory lung disease [10,18,24,25]. In the lungs isolated after 4 days of infection, combination VLPs groups had significantly lesser levels of pro-inflammatory cytokines IFN-γ and IL-6 than Naïve + Challenge group (Fig 6A and 6B). In particular, a significant reduction of inflammatory cytokine IFN-γ in lung was found in the combinatorial VLPs group (Fig 6A).

### Combinatorial VLPs induced significantly higher level of protective efficacy against lethal challenge infection

**Fig 7:**
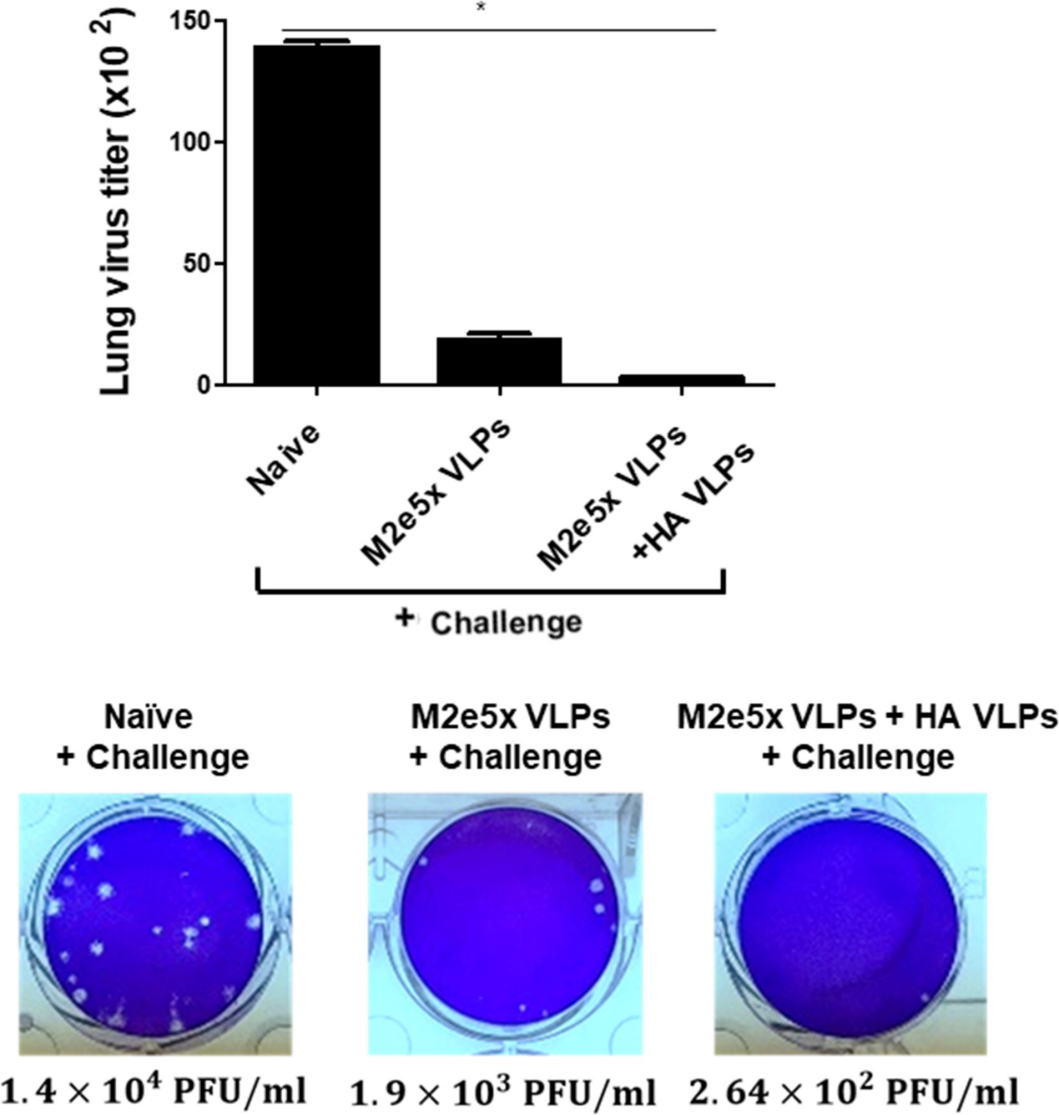
Lung viral titers. Lung samples from individual mouse immunized with VLPs and Naïve + Challenge were collected on day 4 post infection with a lethal dose of A/Viet Nam/1203/2004 (rgH5N1) virus. Significantly lower levels of lung virus loads were found in combinatorial VLPs group compared to M2e5x VLPs group and Naïve + Challenge group (*P < 0.05). Data are expressed as mean ± SD.

**Fig 8:**
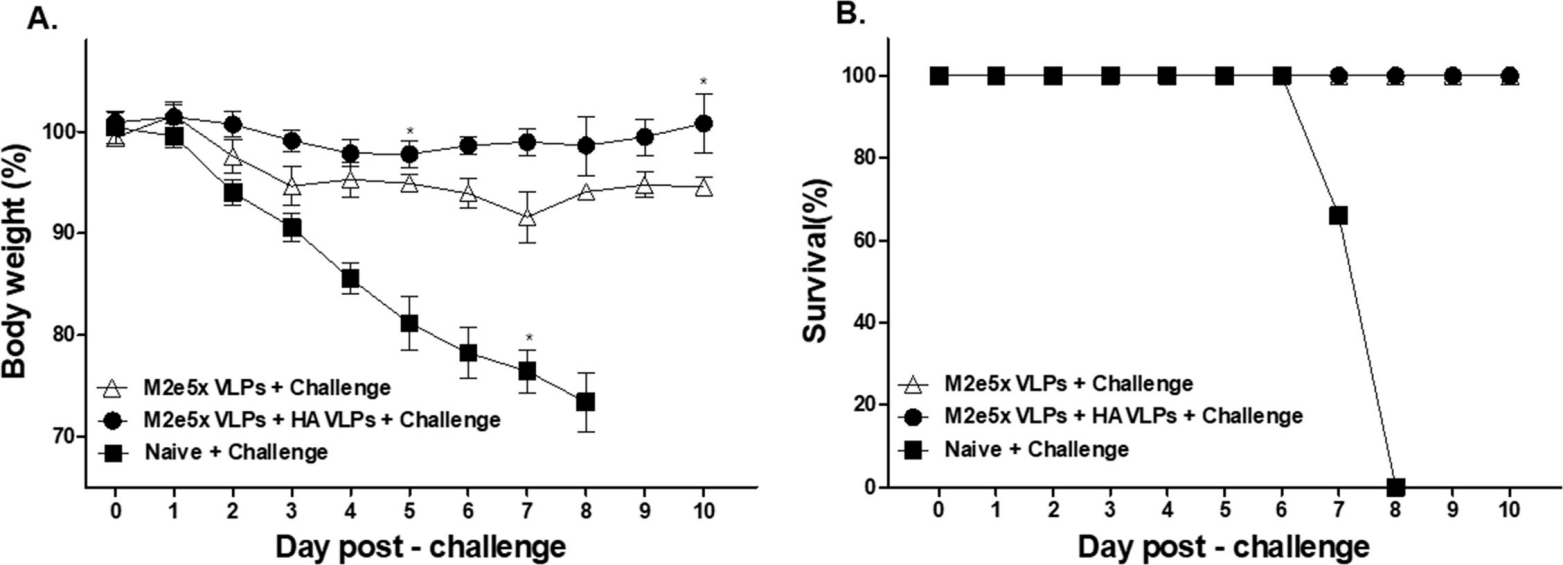
Protection efficacy of mice from lethal influenza virus challenge. Upon intramuscular immunization with VLPs and subsequent challenge infection with a lethal dose of A/Viet Nam/1203/2004 (rgH5N1) virus, mice body weight (A) and survival (B) were monitored daily for 10 days. Combinatorial VLPs showed significantly less body weight loss compared to combination M2e5x VLPs whereas all mice in Naïve + Challenge group reached endpoint. Data are expressed as mean ± SD, *P < 0.05.

At 4 days after challenge with avian influenza A/Viet Nam/1203/2004(rgH5N1), virus titers in lungs were measured. Virus titer in Naive + Challenge was found to be significantly higher than vaccination group (Fig 7). The combination HA VLPs + M2e5x VLPs group showed significantly lower levels of lung virus loads compared to the M2e5x VLPs group. Upon avian influenza A/Viet Nam/1203/2004(rgH5N1) challenge infection, all mice in Naive + Challenge group showed significant body weight loss and died on the 7th day, whereas all mice in combinatorial group survived (Fig 8A and 8B). Importantly, combinatorial VLPs group showed significantly lower body weight loss compared to the M2e5x VLPs group (Fig 8A).

## Discussion

Highly pathogenic avian influenza (HPAI) viruses are responsible for causing high mortality rates and substantial economic losses in poultry industry. However, reassortant influenza vaccines containing HA and NA genes from avian influenza virus cannot provide complete protection in chicken models [26]. Inactivated split vaccine inducing HA immunity of A/Vietnam/1194/04 (clade 1) H5N1 strain was not sufficient for complete protection against heterologous A/Indonesia/5/2005 (clade2) H5N1 virus in ferrets [27]. M2e5x VLPs as a universal vaccine candidate has been reported to induce protection against H3N2 or H5N1 in a mice model [15]. However, M2e5x VLP-immunized mice showed approximately 9% body weight loss. The same M2e5x VLP immunized chickens were not protected against HPAI H5 infection [1]. Thus, it would be desirable to develop an alternative strategy that can significantly improve protection. We hypothesized that M2e5x VLPs in combination with HA VLPs would significantly enhance vaccine efficacy against avian influenza virus infection.

M2e immunity induced by vaccination of mice with M2e5x VLPs have been reported to confer protection via antibody-mediated phagocytosis by macrophage and natural killer (NK) cell-dependent ADCC and HA-specific antibody prevents infection by neutralizing the virus [28]. Thus, in the current study, combinatorial VLPs containing M2e5x VLPs and HA VLPs showed better protection than M2e5x VLPs alone. We observed that combination of VLPs induced higher levels of H5 virus-specific IgG, IgG1, IgG2a and IgA antibody responses in sera or lungs, showing higher ratio of IgG2a/IgG1, compared to M2e5x VLPs. Significantly higher levels of IgG antibody secreting cell responses from spleens and lungs are likely to play a role in the effective viral clearance compared to M2e5x VLPs alone [18]. Since IgG2a isotype is responsible for assisting the clearance of virus-infected host cells and IgG1 isotype antibodies are likely to be involved in neutralizing homologous virus [29], combinatorial VLPs was more effective in lowering lung viral loads and contributed to reducing body weight loss than M2e5x VLPs alone [6].

Mice immunized with either combinatorial VLPs or M2e5x VLPs alone significantly reduced both IFN-γ and IL-6 in lungs compared to Naïve + Challenge control, and the former of the two also displayed significantly lessened lung IFN-γ levels compared to the latter. A significant inverse correlation has been reported between the levels of inflammatory cytokines including IL-6 and IFN-γ and lung viral loads [24]. In our current study, IFN-γ as a pro-inflammatory cytokine showed significantly lower levels in VLP vaccinated mouse groups compared to non-immunized mice which display high lung viral titers, consistent with pro-inflammatory cytokine IL-6. We observed that VLP vaccinated mice showed lower lung virus titers (Fig 7), indicating there is an inverse correlation between lung viral titers and the levels of inflammatory. The results indicated that higher vaccine efficacy from VLP immunization showed lower proinflammatory cytokine response. This is consistent with previous our and other studies demonstrating that immunized mice showing higher efficacy (lower lung viral titers) show lower proinflammatory cytokines (IFN-γ and IL-6) in the lungs of mice than non-immunized mice upon infection with influenza virus [18,25,30]. It is a different measure for infiltration of virus-specific CD8+ T cells into lungs coincided with an increase of pulmonary anti-viral IFN-γ and recovery from viral infection [31]. VLP vaccinated mice showed lower levels of lung virus titers and inflammatory cytokines. This is consistent with previous studies from this and other studies demonstrating that immunized mice showing higher efficacy (high levels of lung viral titers) compared to non-immunized mice upon challenge infection with influenza virus showed lower levels of proinflammatory cytokines (IFN-γ and IL-6) in the lungs [10,18,25]. The previous study demonstrated that high levels of IL-6 and IFN-γ were detected in naive mouse lungs after infection, whereas little or no proinflammatory cytokines were present in the lungs of the influenza HA VLP-immunized mice[10]. Since M2e5x VLPs vaccination has been reported to lower lung inflammatory cytokine IL-6 compared to naïve control [15], inclusion of HA VLPs in the combinatorial VLPs would provide additive protection.

Interestingly, combinatorial VLPs showed high levels of CD8^+^ T cell and CD4^+^ T cell responses, whereas M2e5x VLPs only induced CD4^+^ T cell responses, indicating HA VLPs are more likely to contribute to inducing higher levels of CD8^+^ T cell responses. These findings were consistent with the previous study that reported vaccination with M2e5x VLPs induced low levels of M2e-specific cytokine producing CD8^+^ T cells, similar to those observed in naïve mice after infection [16].

In summary, compared to M2e5x VLPs, combinatorial VLPs vaccination induced higher levels of H5 virus-specific IgG, IgG1, IgG2a and IgA antibody responses in sera or lungs and significantly reduced lung inflammatory responses, which may contribute to significant reduction of lung viral load and enhanced protection. These results indicated that combinatorial VLPs containing M2e5x VLPs and HA VLPs could be a better candidate in inducing improved vaccine efficacy against avian influenza infection.

## Supporting information

S1 Table Humane-endpoints-checklist. (DOCX)
